# Establishment of a reference range for hair cortisol using a HPLC-MS/MS method in a Northern Italian population

**DOI:** 10.1007/s40618-026-02933-0

**Published:** 2026-05-26

**Authors:** Federico Fortuna, Alessia Lanno, Davide Magrin, Amelia Locatelli, Diego Gonzalez, Dario Jacobsen, Juan Jamardo, Bibiana Fabre, Alice Passoni, Renzo Bagnati

**Affiliations:** 1 Facultad de Farmacia y Bioquímica, Departamento de Bioquímica Clínica, Universidad de Buenos Aires, Instituto de Fisiopatología y Bioquímica Clínica (INFIBIOC), Ciudad de Buenos Aires, Argentina; 2 Facultad de Farmacia y Bioquímica, Consejo Nacional de Investigaciones Científicas y Técnicas (CONICET), Ciudad de Buenos Aires, Argentina; 3 Department of Environmental Health Sciences, Istituto di Ricerche Farmacologiche Mario Negri IRCCS, Via Mario Negri 2, Milano, 20156 Italy

**Keywords:** Hair cortisol, Reference range, LC-MS/MS, Hypothalamic-pituitary-adrenal axis (HPA), Chronic stress biomarker

## Abstract

**Purpose:**

Hair cortisol concentration (HCC) represents a promising biomarker for long-term hypothalamic–pituitary–adrenal (HPA) axis activity. However, its routine clinical application is currently limited by the lack of well-established reference ranges. We aimed to establish population-based reference intervals for HCC in healthy adults from Northern Italy, using a liquid chromatography-tandem mass spectrometry (LC-MS/MS) method following the Clinical and Laboratory Standards Institute (CLSI) EP28-A3c guidelines.

**Methods:**

Hair samples were collected from 256 healthy volunteers selected using strict inclusion and exclusion criteria. Cortisol quantification was performed by LC-MS/MS using a deuterated internal standard, guaranteeing high sensitivity and specificity. The method was validated for linearity, limit of detection, extraction efficiency, matrix effect, robustness across different sample weights, and inter-batch reproducibility using an internal quality control sample. Reference intervals were defined nonparametrically (2.5th–97.5th percentiles). Associations with demographic, clinical, and lifestyle variables were assessed.

**Results:**

The established reference interval was 0.84 to 8.07 pg/mg, with a median of 2.41 pg/mg. HCC was significantly associated with body mass index (ρ = 0.160, *p* = 0.010) and weakly correlated with perceived stress (ρ = 0.137, *p* = 0.028). However, no significant associations were observed with sex, age, smoking status, alcohol consumption, physical activity, hair dyeing, or hair washing frequency.

**Conclusion:**

This study provides, for the first time, a population-based reference interval for hair cortisol concentrations using LC-MS/MS in healthy adults. These findings offer a benchmark for future research, supporting the use of HCC as a biomarker in epidemiological and translational research contexts.

**Supplementary Information:**

The online version contains supplementary material available at https://doi.org/10.1007/s40618-026-02933-0.

## Introduction

Cortisol is a key glucocorticoid hormone secreted by the adrenal cortex, and plays a vital role in regulating metabolism, immune function, inflammation, and physiological stress response. Its secretion is modulated by the hypothalamic-pituitary-adrenal (HPA) axis, a neuroendocrine system essential for adapting to stress and homeostasis [1]. Given the importance and diversity of its biological roles, cortisol has become a central biomarker in the evaluation of endocrine disorders and stress-related conditions. Abnormal cortisol levels are indicative of endocrine diseases such as adrenal insufficiency and Cushing’s syndrome [2]. Beyond classical endocrine disorders, persistent dysregulation of the HPA axis due to chronic psychosocial stress has been linked to a wide spectrum of metabolic, immune, and neuropsychiatric disorders [3].

Cortisol levels present short-term fluctuations over minutes to hours due to pulsatile secretion and circadian rhythm [4]. Traditional methods of cortisol quantification involve the analysis of serum, saliva, or urine. Serum and saliva reflect cortisol concentrations at the time of sampling, while urine typically reflects integrated cortisol secretion over a collection period of up to 24 h. While suitable for evaluating cortisol levels in pathologies such as Cushing’s syndrome or Addison’s disease, these matrices do not capture long-term cortisol exposure, which is crucial for understanding the impact of chronic stress [5].

In this context, alternative matrices have been explored, with hair emerging as one of the most promising options. Since the first report on hair cortisol measurement in 2004 [6], interest has grown rapidly due to its non-invasive sampling, intraindividual stability, and ability to reflect systemic cortisol exposure over several months [7, 8].

Cortisol incorporates into the hair shaft mainly through passive diffusion from blood at the follicular level during the anagen phase, with additional contributions from sweat and sebaceous secretions [9, 10]. The posterior vertex region is preferred for sampling due to its high proportion of anagen-phase follicles and lower variability [11]. Assuming an average growth rate of 1 cm per month, segmental analysis allows for retrospective reconstruction of hormone exposure, an assumption validated across different epidemiological studies [12–14]. Another key advantage of hair cortisol is its independence from diurnal fluctuation and acute stressors, allowing for retrospective quantification and providing a diagnostic tool that reflects systemic cortisol levels over time [15]. Beyond its biological stability, hair can be stored at room temperature for extended periods, and samples can be collected by non-medical personnel, which facilitates large-scale population-based studies [16]. However, emerging evidence suggests that HCC may be subject to seasonal shifts, an issue that should be considered or recorded when evaluating long-term endocrine output [17].

For hair cortisol quantification, immunoassays—initially adapted from saliva analysis—were widely used [18]. More recently, liquid chromatography coupled to tandem mass spectrometry (LC-MS/MS) has become the gold standard method, overcoming traditional limitations of immunoassays such as antibody cross-reactivity. This is particularly relevant when quantifying cortisol at the low concentrations found in hair and has improved comparability across studies [19–21].

Beyond analytical advantages, clinical interest in hair cortisol is increasing. Elevated levels have been reported in Cushing’s syndrome, major depression, cardiovascular disease, diabetes, obesity, and chronic pain, highlighting its potential for diagnosis, prognosis, and monitoring [22–24].

Despite these advances, a major limitation to clinical translation is the absence of standardized reference intervals in healthy adults. Most studies report mean values or ranges in convenience samples, often using immunoassay-based methods. However, no study has yet established a population-based reference interval for hair cortisol in healthy adults following the Clinical and Laboratory Standards Institute (CLSI) EP28-A3c guidelines, which define specific criteria for reference population selection and minimum sample size. As a result, the interpretation of results across cohorts and their application in diagnostic frameworks remain challenging.

Therefore, the aim of this study was to establish a population-based reference interval in healthy adults in a Northern Italian Population, in accordance with the CLSI EP28-A3c guidelines [25] using an LC-MS/MS-based method for hair cortisol quantification.

## Materials and methods

### Study population and epidemiological data

A total of 270 apparently healthy individuals were recruited from the academic and research community of the “Istituto di Ricerche Farmacologiche Mario Negri” IRCCS (Milan, Italy) for this study. Eligible participants were of any gender, aged between 18 and 75 years, and were included only after signing the informed consent and voluntarily agreeing to participate. The population included researchers, PhD and undergraduate students, and administrative staff.

Participants were considered healthy based on their responses to a self-administered screening questionnaire. Although this approach may overlook some undiagnosed or subclinical conditions, it is a practical and widely accepted method for reference population selection in large-scale studies, in accordance with CLSI EP28-A3c recommendations [25]. To be included, individuals had to certify that they did not suffer from any condition that could influence glucocorticoid levels, including endocrine disorders (such as Addison’s disease or Cushing’s syndrome) or psychiatric disorders (such as anxiety, depression, obsessive-compulsive disorder, or attention deficit disorders). Additionally, participants had to confirm that they were not undergoing treatment with medications known to affect cortisol levels, including corticosteroids (regardless of route of administration) or psychiatric/neurological drugs, such as antipsychotics, antidepressants, anxiolytics, or hypnotics. Exclusion criteria comprised any current or past diagnosis of endocrine or psychiatric conditions that could interfere with cortisol regulation, as well as any treatment that might influence the HPA axis. Specifically, this included patients undergoing psychiatric or chemotherapy treatments, or any chronic therapy with potential effects on the endocrine system or glucocorticoid metabolism. Pregnant women were excluded from the study, as well as individuals with less than 2 cm of hair. Given that recruitment occurred during regular daytime hours at the research institute, shift workers were not included.

All participants completed a standardized health questionnaire adapted from the CLSI EP28-A3c sample model, provided in the Supplemental Materials. The adaptation specifically involved the inclusion of dedicated items to assess hair-related habits known to potentially influence cortisol levels, such as the use of specific hair products (e.g., shampoo and conditioner), hair dye usage, and weekly washing frequency. Additionally, the questionnaire collected comprehensive data on demographics (age, height, weight, and occupation), general health status, chronic conditions, recent hospitalizations, and current prescribed medications. Lifestyle factors such as physical exercise, tobacco use, and alcohol consumption were also recorded.

In addition, participants completed the Cohen Perceived Stress Scale (PSS-10) [26]. The PSS-10 is a widely used 10-item instrument designed to assess the extent to which individuals perceive situations in their lives as stressful, answered on a 5-point Likert scale ranging from “never” to “very often”.

The study was conducted in accordance with the Declaration of Helsinki and approved by the local Ethics Committee (Comitato etico territoriale Lombardia 6), protocol number 0026526/25, on 13/06/2025. Written informed consent was obtained from all participants prior to sample collection.

### Reagents and chemicals

Isopropanol and methanol were purchased from Carlo Erba and were LC-MS grade. Ultrapure water was obtained from a Milli-Ro 60 Water System, Millipore, Milford, MA, USA. Ammonium formate (purity ≥ 99%) was purchased from Avantor. Hydrocortisone (purity ≥ 98%) and hydrocortisone-D_4_ (purity ≥ 99%) were purchased from Cabru. Hydrocortisone and hydrocortisone-D_4_ were prepared as stock solutions in methanol (MeOH) at the concentration of 1 mg/mL. The stock solution of hydrocortisone was diluted in MeOH to make a series of six working solutions for constructing calibration curves, validation and sample analyses. Stock solutions of the standards were kept at -20 °C prior to analysis.

### Hair sample collection and cortisol extraction

Hair samples were collected using scissors from the posterior vertex of the scalp, as close to the scalp as possible. Considering that hair grows approximately 1 cm per month, 3 cm segments were collected to assess cortisol levels over the past three months [14]. Hair samples were collected during spring to ensure seasonal consistency across the cohort and to account for potential chronobiological variations in hair cortisol concentrations [17]. Each sample was stored in a paper envelope at room temperature until analysis.

To remove external contaminants, a two-step wash with 5 mL of isopropanol was performed. This is a standard procedure that effectively removes surface contamination while preserving endogenous cortisol [11, 27]. The hair was then cut into small pieces with scissors, and 25 mg were weighed into a glass tube. Based on our robustness experiments, this sample weight was selected as a practical balance between ensuring reliable quantification and feasibility for samples with limited hair availability. Samples were spiked with cortisol-D₄ at a concentration of 20 pg/mg as internal standard (IS), and 4 mL of methanol were added. Tubes were sealed and incubated in a shaker at room temperature for 3 h, followed by overnight incubation at 50 °C for steroid extraction to maximize the cortisol extraction [27].

After incubation, the methanol extract was collected in clean glass tubes and evaporated to dryness under a stream of nitrogen. The residue was reconstituted in 200 µL of methanol and vortexed for 1 min. After centrifugation to allow particulate matter to settle, the supernatant was transferred to a plastic vial with a micro-insert, and 4 µL were injected into a liquid chromatography-tandem mass spectrometry (LC-MS/MS) system for cortisol determination.

### Chromatographic and mass spectrometric conditions

Analyses were performed using an API 5500 triple quadrupole mass spectrometer (Sciex, Framingham, MA, USA) operating in multiple reaction monitoring (MRM) mode, coupled to a 1260 Infinity II high-performance liquid chromatography (HPLC) system (Agilent Technologies, US). The mass spectrometer was equipped with an electrospray ionization (ESI) source operating in negative ion mode. Chromatographic separation was obtained on a Robusta C18 column (100 × 2.1 mm, 3 μm; SepaChrom), maintained at 35 °C with a flow rate of 0.2 mL/min. The mobile phase consisted of 10 mM ammonium formate in water (solution A) and methanol (solution B). The following gradient elution program was used: from 1% B to 99% B in 12 min, maintained 6 min, returned to 1% B in 1 min, followed by 6 min of re-equilibration. For both the analyte and the IS, one quantifier and one qualifier transition were monitored. Specifically, for cortisol, the selected transitions were *m/z* 331.1–282.1 (quantifier) and *m/z* 331.1–297.1 (qualifier). For the IS, the transitions were *m/z* 335.2–286.1 (quantifier) and *m/z* 335.2–301.1 (qualifier).

### Method development and validation

The method was validated in terms of linearity range, limit of detection (LOD), lower limit of quantification (LLOQ), extraction efficiency and matrix effect in accordance with the principles of the ICH M10 guideline on bioanalytical method validation [28].

***LOD***,*** LLOQ***,*** linearity***,*** and inter-batch reproducibility***

LOD was set at a signal-to-noise ratio (S/N) equal to 3. Due to cortisol’s endogenous nature in hair, a completely blank matrix was unavailable; thus, the S/N was calculated using the transition of the deuterated IS corresponding to cortisol’s quantifier transition. The IS therefore served as a surrogate for the analyte, based on the assumption that the deuterated compound exhibits fully overlapping chemical behavior and instrumental response with the parent compound. The S/N was determined at two IS concentrations (3.1 pg on-column and 14.7 pg on-column), each analyzed in six replicates. Average S/N values were calculated for each concentration, and LOD was extrapolated proportionally to S/*N* = 3. This provides an indirect estimate rather than direct experimental verification with low-level spikes.

LLOQ was set at a S/N ratio over than 5 and according to guidelines with the acceptance criteria for precision (CV% < 20%) and accuracy (within ± 20% of the nominal value).

Linearity was evaluated over the concentration range of 0–150 pg/mg, using eight calibration points (0, 0.45, 1.5, 3, 10, 30, 100, and 150 pg/mg). The IS concentration was 20 pg/mg. Calibrators were prepared by spiking standard solutions into methanol, and the calibration procedure was repeated across five independent analytical batches. Calibration curves were constructed by plotting the peak area ratio of cortisol to the IS against nominal concentrations. Linear regression analysis with a 1/x² weighting factor was applied, to minimize the influence of higher-concentration standards and ensure maximum accuracy at the lower concentrations of the calibration curve, where most samples are expected [29] .The quality of the calibration curves was assessed by examining the determination coefficient (r²) and the mean accuracy of calibrators both within and across analytical sessions.

To evaluate the reproducibility of the method across different analytical sessions, a pooled hair sample was prepared from a full-scalp shave of a single donor. This approach was necessary because a single vertex sample does not provide sufficient hair mass for independent extraction across multiple batches. Moreover, due to the endogenous nature of cortisol, we were not able to use spiked quality control (QC) at different levels. The pooled sample was thoroughly homogenized and analyzed in 13 independent analytical batches throughout the study period. Note that this does not constitute full QC-based validation per ICH M10 guidelines, which recommend independent QC samples at multiple concentrations; however, it provides a practical assessment of inter-batch reproducibility in this challenging matrix.

***Extraction efficiency and matrix effect***

Extraction efficiency was assessed by comparing: (A) the peak area of cortisol and IS spiked into the matrix before extraction, and (B) the peak area of cortisol and IS spiked into the matrix after extraction. At least five replicates of QC samples were analyzed at two concentration levels: low QC (LQC, 1.35 pg/mg) and high QC (HQC, 135 pg/mg). The mean extraction efficiency at each QC level was required to have a coefficient of variation (CV) ≤ 20%.

Matrix effect refers to the influence of co-eluting substances from the biological matrix on the analyte signal. To quantify this, the matrix factor (MF) was calculated using five QC samples at the two concentration levels (1.35 and 135 pg/mg). For both the analyte and the IS, MF was determined as the ratio of (B) the peak area in the presence of matrix (spiked into extracted blank matrix post-extraction) to (C) the peak area in the absence of matrix (pure standard solution). A MF < 1 indicates ion suppression, while MF > 1 indicates ion enhancement. To correct for IS variability, the normalized matrix factor was calculated as the ratio of the analyte MF to the IS MF. The CV of normalized MFs at each concentration level was required to be ≤ 15%, in accordance with validation guidelines [28].

***Evaluation of Sample Weight***

In order to evaluate the robustness of the extraction protocol with varying sample weights, three independent hair samples from different subjects (A, B, and C) were extracted in triplicate using 10 mg, 25 mg, and 50 mg of matrix.

### Reference range

Reference intervals were calculated in accordance with the CLSI guideline EP28-A3c for defining and establishing reference intervals in the clinical laboratory [25]. Following the clinical approach recommended by the guideline, the reference interval is defined as the range between two values, namely, a lower and upper reference limit, which are estimated to include a specified percentage, typically 95%, of the values from a reference population. These limits correspond to the 2.5th and 97.5th percentiles of the distribution of test results obtained from the reference sample, thus capturing the central portion of the population’s physiological variation.

### Statistical methods

Statistical analyses were conducted using SPSS software (version 23.0, IBM Corp., Armonk, NY, USA). Data distribution was assessed using the Kolmogorov–Smirnov test. Continuous variables are expressed as mean ± standard deviation (SD) for normally distributed data, or as median and range for non-normally distributed data. Hair cortisol concentrations showed a non-normal distribution, as confirmed by the Kolmogorov–Smirnov test. To allow the use of parametric tests (t-tests, ANOVA), which offer greater statistical power than their non-parametric alternatives, values were natural log-transformed (ln), resulting in a normal distribution. The reference interval, however, was calculated using raw (untransformed) values and defined by the 2.5th and 97.5th percentiles, as this approach is distribution-independent and provides clinically interpretable limits. Differences between two groups were evaluated using independent samples *t*-tests or Mann–Whitney U tests, as appropriate. One-way ANOVA was used to compare continuous variables across more than two groups when assumptions of normality and homogeneity of variance (assessed by Levene’s test) were met; otherwise, the non-parametric Kruskal–Wallis test was applied.

Correlations between continuous variables were assessed using Spearman’s rank correlation coefficient. A two-tailed p-value < 0.05 was considered statistically significant.

## Results

### Method development and validation

***LOD***,*** LLOQ***,*** linearity***,*** and inter-batch reproducibility***

The estimated LOD was 0.60 pg on-column (S/*N* = 3), based on IS signal extrapolation. This sensitivity level confirms the method’s suitability for detecting and quantifying cortisol in hair samples.

The back-calculated concentrations of calibration standards were within ± 15% of the nominal values for all levels, also for the LLOQ, set at 0.45 pg/mg. The precision, expressed as CV%, was below the 10% for all points, including LLOQ, indicating high repeatability of the calibration model. Table 1 reports the results obtained from the five calibration curves analyzed in different analytical samples and the calculated parameters. These results confirm the accuracy and precision of the calibration curve across the analytical range.

**Table 1:** Linearity range of cortisol (0.45, 1.5, 3, 10, 30, 100, and 150 pg/mg) was evaluated across five independent analytical sessions. Results are presented as mean, standard deviation (SD), accuracy %, coefficient of variation %

Nominal concentration (pg/mg)	Calculated concentration (pg/mg)					Mean	SD	Acc. %	CV %
**0.45**	0.449	0.438	0.454	0.44	0.439	0.444	0.007	99%	2%
**1.5**	1.46	1.58	1.53	1.59	1.63	1.558	0.065	104%	4%
**3**	3.25	3.29	2.72	3.16	3.07	3.098	0.227	103%	7%
**10**	9.49	9.33	9.99	9.62	9.2	9.526	0.304	95%	3%
**30**	29.6	29.00	27	27	29.6	28.44	1.337	95%	5%
**100**	93.7	99.60	109	104	95.1	100.28	6.333	100%	6%
**150**	161	148.00	161	152	159	156.2	5.890	104%	4%

Linear regression parameter									
**Slope**	0.0997	0.109	0.093	0.101	0.0881	0.0982	0.007		
**r** ^**2**^	0.9980	0.9981	0.9966	0.9980	0.9979	0.9977	0.0006		

The slope values of the five calibration curves ranged from 0.0881 to 0.109, with a mean of 0.0982 and a standard deviation of 0.0070, indicating good consistency in the analytical response (Table 1). The coefficient of determination (R²) was ≥ 0.9966 for all calibration curves, with a mean R² of 0.9977 (SD = 0.0006), demonstrating excellent linearity across the calibration range.

Inter-batch reproducibility was assessed using a pooled hair sample analyzed across 13 independent batches. The mean cortisol concentration was 6.51 pg/mg with a CV of 19%, demonstrating acceptable reproducibility (Table S1). This CV reflects the intrinsic heterogeneity of whole-scalp hair compared to a standardized vertex sample, yet demonstrates method robustness over extended use.

***Extraction efficiency***,*** and matrix effect***

As shown in Table 2, the absolute extraction efficiency of cortisol was 86% (CV% = 11%) at the LQC level (1.35 pg/mg) and 77% (CV% = 9%) at the HQC level (135 pg/mg), indicating acceptable and consistent extraction efficiency across the calibration range. The IS (Cortisol-D_4_) showed an extraction efficiency of 117% (CV% = 15%) at LQC and 77% (CV% = 9%) at HQC.

**Table 2:** Extraction efficiency of cortisol and cortisol-D_4_ calculated analyzing five quality control samples at two concentration levels: LQC (1.35 pg/mg) and HQC (135 pg/mg). Results are presented as mean, standard deviation (SD) and coefficient of variation %

Quality control level (pg/mg)	Cortisol Extraction efficiency			Cortisol-D_4_ Extraction efficiency		
	Mean	±SD	CV%	Mean	±SD	CV%
1.35	86%	10%	11%	117%	17%	15%
135	77%	7%	9%	77%	7%	9%

Regarding the matrix effect, as reported in Table 3, we obtained a normalized MF close to 1 for the LQC and HQC. The CV% was below 15% for the HQC, and at this threshold for the LQC, likely due to the higher contribution of endogenous cortisol at low concentrations.

**Table 3:** Matrix effect for cortisol and cortisol-D_4_ calculated analyzing five quality control samples at two levels: LQC (1.35 pg/mg) and HQC (135 pg/mg). Results are presented as matrix factor for analyte and internal standard with mean, standard deviation (SD) and coefficient of variation %. The normalized matrix factor obtained as ratio of the two matrix factors is also reported as mean, SD, and coefficient of variation %

Quality control level (pg/mg)	Cortisol matrix factor			Cortisol-D_4_ matrix factor			Normalized matrix factor		
	Mean	±SD	CV%	Mean	±SD	CV%	Mean	±SD	CV%
1.35	1.28	0.06	7%	1.29	0.17	13%	1.01	0.15	15%
135	0.74	0.07	9%	0.76	0.08	11%	0.98	0.07	7%

In order to evaluate the robustness of the extraction protocol, cortisol was quantified using three different sample weights (10 mg, 25 mg, and 50 mg) from three independent subjects (A, B, and C). Cortisol concentrations obtained across the different weights showed good consistency within each subject. The calculated mean concentrations were 1.29 pg/mg (CV% = 16%) for Subject A, 1.83 pg/mg (CV% = 8%) for Subject B, and 7.56 pg/mg (CV% = 11%) for Subject C. These results confirm that the method provides reproducible quantification of cortisol even when only 10 mg of hair is available, supporting its applicability to samples with limited material.

### Reference value

A total of 270 volunteers were recruited for this study. Among these, 11 were excluded because they did not meet the inclusion criteria, and 3 were excluded for not completing the self-administered health questionnaire. The final study population consisted of 256 participants.

Table 4 presents the sociodemographic characteristics of the analyzed population and the median and observed range (minimum–maximum) of hair cortisol concentrations and self-reported perceived stress scores. The reference interval was subsequently calculated from this dataset.

**Table 4:** Descriptive Characteristics of the Study Population (*n* = 256)

Variable	Results
Age, mean ± SD Gender, % (n) BMI, median (range) Physical Activity, % (n) Smoker, % (n) Alcohol Consumption, % (n) Chronic Illness, % (n) Dyed hair, % (n) Perceived stress, median (range) Hair cortisol, median (range) **Profession/Occupation** % (n) Researcher PhD student Bachelor student Administrative staff Other*	36 ± 14 years F: 67.2 (172); M: 32.8 (84) 22 (17–40) kg/m2 Yes: 61 (155) No: 39 (101) Yes: 18 (46) No: 82 (210) Yes: 71 (181) No: 29 (75) Yes: 14 (37) No: 86 (219) Yes: 27 (69) No: 73 (187) 18 (0–34) 2.41 (0.48–15.40) pg/mg 31 (80) 14 (36) 21 (53) 25 (64) 9 (23)

F: female; M: male. Results are expressed as mean ± standard deviation (SD) or median (range), according to the data distribution. *Other included retirees, schoolteachers, engineers, laboratory technicians, a psychologist, and a nurse

The distribution of hair cortisol values was non-parametric, as confirmed by the Kolmogorov–Smirnov test (K-S D = 0.151, *p* < 0.001). Therefore, the reference range was defined using the 2.5th and 97.5th percentiles, yielding a population-based reference range of 0.84 to 8.07 pg/mg.

In order to allow the use of parametric statistical tests, a natural logarithmic transformation (ln) was applied to cortisol values resulting in a normal distribution (K-S D = 0.037, *p* = 0.200).

When evaluating the difference in hair cortisol levels by gender, an independent samples *t*-test was performed with no significant differences found between females and males (*p* = 0.468), indicating similar hair cortisol levels across both groups.

Similar analyses were conducted to examine differences in hair cortisol levels according to smoking status, alcohol consumption, regular physical activity, hair dyeing, and presence of chronic illness. Independent samples *t*-tests performed revealed no significant differences between groups for any of these variables (*p* > 0.05 for all comparisons, Table 5).

**Table 5:** Group comparisons of hair cortisol levels (independent t-tests)

Variable	Groups Compared	Test Statistic (t)	Degrees of freedom	*p*-value
Gender	Female vs. Male	-0.727	254	0.468
Tobacco use	Yes vs. No	0.770	254	0.442
Alcohol Consumption	Yes vs. No	-0.537	254	0.591
Physical Activity	Yes vs. No	-1.576	254	0.116
Hair Dyeing	Yes vs. No	0.960	254	0.339
Chronic Illness	Yes vs. No	0.436	254	0.565

Then, to assess potential differences in hair cortisol levels across multiple categories, one-way ANOVA tests were conducted (Table 6). Specifically, comparisons were made across age groups, frequency of physical activity, frequency of alcohol consumption, frequency of hair washing, occupation and body mass index (BMI) categories. In all cases, no statistically significant differences in ln-transformed hair cortisol levels were observed between the groups (*p* > 0.05), except for BMI, where a statistically significant difference was found (*p* = 0.024).

**Table 6:** One-way ANOVA results comparing ln-transformed hair cortisol levels across categorical variables

Variable	Groups Compared	Degrees of freedom (between; within)	F	*p*-value
Age groups	4	3;252	0.167	0.919
BMI categories	4	3;252	2.854	0.024
Alcohol Consumption^a^	4	3;177	0.801	0.495
Physical Activity^b^	4	3;151	2.075	0.106
Hair washing^c^	5	4;157	0.216	0.929
Occupation	5	4;252	0.704	0.590

Age groups: 18–29, 30–44, 45–59, ≥ 60. BMI categories: underweight (< 18.5), normal weight (18.5–24.9), overweight (25–29.9), obese (≥ 30). Alcohol consumption frequency: 1 dose/week, 2 doses/week, 3 doses/week, > 3 doses/week. Physical activity frequency: 30–60 min/week, 1–3 h/week, 3–5 h/week, ≥ 5 h/week. Hair washing frequency: 1 time/week, 2 times/week, 3 times/week, 4 times/week, ≥ 5 times/week. Occupation: Researcher, PhD Student, Bachelor Student, Administrative Staff, Other. ^a^Analysis performed only on participants reporting alcohol consumption (*n* = 181). ^b^Analysis performed only on participants reporting physical activity (*n* = 155). ^c^ Analysis performed only on participants for whom hair washing frequency data were available (*n* = 162)

Subsequently, the sample was divided into two groups based on scores from the Perceived Stress Scale: individuals scoring at or above the 75th percentile (23 points) were classified as experiencing high perceived stress, while the remainder were categorized as lower stress. This stratification aimed to assess potential associations between perceived stress levels and variations in hair cortisol concentrations. However, no significant differences were observed between these groups (t(254) = -1.645, *p* = 0.081).

In addition, to facilitate clinical interpretation and provide a direct overview of raw cortisol values (pg/mg), the median and interquartile range (IQR) for these groups are provided in the Supplemental Materials (Tables S2 and S3).

Finally, in order to explore potential associations between perceived stress, physiological stress markers, and demographic or clinical characteristics, Spearman’s rank correlation analyses were conducted. The results revealed a statistically significant moderate inverse correlation between age and perceived stress (ρ = -0.235, *p* < 0.001), indicating that older participants tended to report lower perceived stress levels. Additionally, weak but statistically significant positive correlations were found between perceived stress and hair cortisol levels (ρ = 0.137, *p* = 0.028), as well as between BMI and hair cortisol concentrations (ρ = 0.160, *p* = 0.010). These findings suggest subtle associations between subjective stress perception, biological stress indicators, and participant characteristics.

## Discussion

The method developed in this study aligns with the established framework for hair cortisol quantification, aimed at evaluating the relationship between cortisol levels and chronic stress [30]. Although immunoassay-based techniques remain the most commonly employed due to their low cost and operational simplicity [31], we selected LC–MS/MS for method development owing to its superior sensitivity, precision, and specificity that allow detection of extremely low concentrations [32]. The chromatographic conditions were optimized to ensure adequate separation and analytical robustness. The relatively long run time (~ 25 min) was chosen to maintain selectivity and flexibility for potential future expansion of the method to additional analytes, and is therefore more suited to research applications than high-throughput routine clinical workflows. The detection of endogenous metabolites is normally more laborious than exogenous molecules [33]. For that reason, to ensure the correct quantification of cortisol concentration, we chose to evaluate extraction efficiency and matrix effect as validation parameters. However, the validation of method for quantification of endogenous compounds is challenging, due to the absence of true blanks samples used for quality control preparation. The values obtained for these parameters were consistent with those previously reported in the literature [34–36]. Specifically, normalized matrix factors near unity confirm that the IS effectively compensated for matrix effects, ensuring reliable quantification. Minor deviations in recovery at low concentration levels are expected when working near endogenous background levels. Additionally, inter-batch reproducibility was assessed using an in-house pooled sample (full-scalp shave from a single donor) analyzed across 13 analytical batches, yielding a CV of 19%. While satisfactory for this complex biological matrix, we acknowledge that using a single-donor pool does not fully align with ICH M10 guidelines, which recommend independent QC samples at multiple levels from different matrices or donors. This limitation stems from cortisol’s endogenous nature, precluding reliable spiked QC preparation, and the insufficient mass from vertex samples for multi-batch testing. Future optimizations could explore valid alternatives to assess reproducibility.

To the best of our knowledge, this is the first study to establish a true population-based reference range for hair cortisol concentrations in healthy adults using LC-MS/MS. Some previous studies have proposed reference values, but they are typically based on immunoassays and smaller sample sizes [30, 31, 37]. By contrast, the present study follows the recommendations of the Clinical and Laboratory Standards Institute (CLSI) EP28-A3c guideline for defining reference intervals, not only in terms of analytical method and sample size, but also in the definition of a reference population [25].

When compared to values reported for control groups in other LC-MS/MS-based studies, our results show strong concordance. Staufenbiel et al. found a median of 3.18 pg/mg (IQR: 2.16–5.58) in a sample of 760 individuals, even though no reference range was defined [38]. Other studies, such as Wang et al., reported similar ranges (1.7–9.1 pg/mg) but were based on small control groups [36]. Finally, Wester et al., reported a log-transformed approach in a similar-sized sample (*n* = 266), obtaining a reference interval of 0.68 to 10.49 pg/mg and a geometric mean of 2.67 pg/mg [39]. Although the statistical methods differ (percentiles in our study versus log-transformed mean ± 1.96 SD in theirs), the overall cortisol concentration ranges are consistent with our findings.

Subsequently, we examined potential sources of variation in HCC (Tables 5 and 6). None of these variables showed statistically significant differences in cortisol levels (all *p* > 0.05). Existing literature offers heterogeneous findings regarding the influence of these variables on hair cortisol concentrations (HCC). For instance, in terms of sex differences, several studies reported higher HCC in men compared to women [40–42]. In contrast, our findings align with investigations that identified no significant sex-related differences in healthy adults [43]. In our population, men showed a slightly higher median than women, consistent with the unadjusted observations of Staufenbiel et al. [38], although this trend did not reach statistical significance.

Regarding age, several studies have informed modest positive associations between age and HCC, particularly when examining wide age ranges or older adults [40–42]. De Kruijff et al., have determined reference intervals for HCC by LC-MS/MS for healthy children considering these variations [44].

However, not all studies have confirmed this trend, and many found no association between age and HCC [43, 45, 46]. Our findings align with this latter group, likely because our sample consisted primarily of young and middle-aged adults, potentially limiting the ability to detect age-related effects observed in older cohorts.

When it comes to lifestyle variables such as smoking, alcohol consumption, and physical activity; most studies have found no significant associations with HCC [14, 38, 42]. Consistent with this, our results showed no significant differences between groups suggesting that, habits may not substantially influence long-term hair cortisol accumulation in healthy adults.

Similarly, hair treatments, including washing frequency and the use of dyes or chemical products are often considered potential confounders [47, 48]. Even though some studies have suggested that higher washing frequency might reduce cortisol levels due to repeated exposure to water and shampoo [40], our findings align with more recent investigations that failed to confirm this effect [49, 50]. These results support the view that routine hair care do not substantially affect hair cortisol levels.

Considering occupational groups, hair cortisol concentrations did not differ significantly. This lack of variation is likely due to the overall homogeneity of the work environment in our sample, as all participants were associated with the academic or research field.

No significant differences in HCC were found between individuals with chronic health conditions - such as hypertension, cardiovascular disease or diabetes- and those without. However, given the low prevalence of these conditions in our sample and the binary classification used (presence vs. absence), specific conclusions regarding the impact of chronic illness cannot be drawn.

In addition, BMI was the only variable in our analysis that showed a statistically significant association with hair cortisol concentrations (*p* = 0.024). However, this finding should be treated with caution due to the limited number of obese participants (*n* = 10). Despite this limitation, the higher cortisol levels in this group are consistent with previous evidence suggesting that obesity may reflect a state of chronic HPA axis dysregulation [51, 52]. Similarly, data from the ELSA-Brazil cohort showed a positive association between hair cortisol and weight status [53]. Mechanistically, excess adipose tissue may impair the negative feedback regulation of the HPA axis, while also promoting local cortisol regeneration through increased activity of the enzyme 11β-HSD1. These alterations can lead to sustained cortisol exposure. Nonetheless, the biological mechanisms underlying this relationship are not yet fully understood and require further investigation [51, 54, 55].

We conducted an exploratory evaluation of perceived stress to investigate potential associations with HCC. While the primary goal of this study was to establish reference values for HCC in a healthy adult population, this secondary analysis was included to explore whether subjective stress perception relates to hair cortisol levels. No significant differences in HCC were observed between high and low perceived stress groups based on PSS scores. These findings align with previous studies that failed to identify clear HCC distinctions using subjective stress thresholds [46, 56] suggesting that perceived stress alone may not be sufficient to distinguish individuals with elevated HCC.

However, when treated as a continuous variable, a weak positive correlation between perceived stress and HCC emerged, suggesting a modest association with cumulative cortisol exposure [57].

Finally, a moderate inverse correlation was observed between age and perceived stress levels, suggesting that older individuals may report lower levels of subjective stress. This pattern has been also noted in previous studies, potentially reflecting greater emotional regulation or resilience with aging [58, 59].

This study has several limitations that should be considered. First, the health status of participants was assessed only through a self-administered questionnaire. This approach is practical in large-scale studies, but it does not allow the detection of undiagnosed or subclinical conditions that could influence cortisol levels. Second, although the reference sample was selected in line with CLSI recommendations, most participants were young adults working or studying in the research environments. While this enhances sample homogeneity and internal validity, it may also narrow interpretation when considering populations with different sociodemographic backgrounds. Additionally, all samples were collected in spring. While this ensures internal consistency by eliminating seasonal confounding, it remains to be determined whether such variations reach clinical significance or warrant season-specific reference intervals. Finally, even though LC-MS/MS is a highly accurate and specific method, certain steps such as sample preparation and calibration can vary across laboratories. Although initial efforts toward standardization have been made [21], certified reference materials for hair cortisol are not yet available, and the absence of a standardized protocol remains a challenge for comparing findings across studies [60]. Future multicenter studies using standardized protocols are needed to further validate and extend these reference values in more diverse populations.

In summary, establishing a population-based reference interval provides the baseline that was missing for hair cortisol research. Using a specific and reliable method like LC-MS/MS turns hair cortisol into a practical tool for studying long-term HPA axis activity, especially in clinical and research settings where chronic dysregulation plays a central role.

## Conclusion

This study establishes, for the first time, a population-based reference interval for hair cortisol concentrations in healthy adults using LC-MS/MS, following CLSI guidelines. The defined range (0.84–8.07 pg/mg) provides a reliable benchmark for future research and potential clinical applications involving long-term HPA axis activity.

## Supplementary Information

Below is the link to the electronic supplementary material.

Supplementary Material 1

## Data Availability

The datasets generated during and/or analyzed during the current study are available from the corresponding author on reasonable request.

## Abbreviations

BMI: Body mass index
CLSI: Clinical and laboratory standards institute
CV: Coefficient of variation
ESI: Electrospray ionization
F: Female
HCC: Hair cortisol concentrations
HPA: Hypothalamic-pituitary-adrenal
HPLC: High-performance liquid chromatography
HQC: High quality control
IQR: Interquartile range
IS: Internal standard
K-S: Kolmogorov–smirnov
LC-MS: Liquid chromatography-mass spectrometry
LC-MS/MS: Liquid chromatography coupled to tandem mass spectrometry
LLOQ: Lower limit of quantification
ln: Natural log-transformed
LOD: Limit of detection
LQC: Low quality control
M: Male
MF: Matrix factor
MeOH: MeOH
MRM: Multiple reaction monitoring
PSS-10: Perceived stress scale (10-item)
QC: Quality control
R²: Coefficient of determination
S/N: Signal-to-noise ratio
SD: Standard deviation
