# Supplementary material for: Establishment of a reference range for hair cortisol using a HPLC-MS/MS method in a Northern Italian population
Source: J Endocrinol Invest. 2026 May 26;49(10):2735–46. doi: 10.1007/s40618-026-02933-0 (PMC13627224; doi:10.1007/s40618-026-02933-0)
Supplement: Supplementary file 1 [file 40618_2026_2933_MOESM1_ESM.docx]

**Journal of Endocrinological Investigation**

**Establishment of a Reference Range for Hair Cortisol Using a HPLC-MS/MS Method in a Northern Italian Population**

Federico Fortuna^1,2^, Alessia Lanno^3^, Davide Magrin^3^, Amelia Locatelli^3^, Diego Gonzalez^1^, Dario Jacobsen^1^, Juan Jamardo^1^, Bibiana Fabre^1^, Alice Passoni^3, *^, Renzo Bagnati^3^

^1^Universidad de Buenos Aires, Facultad de Farmacia y Bioquímica, Departamento de Bioquímica Clínica, Instituto de Fisiopatología y Bioquímica Clínica (INFIBIOC), Ciudad de Buenos Aires, Argentina

^2^Consejo Nacional de Investigaciones Científicas y Técnicas (CONICET), Facultad de Farmacia y Bioquímica, Ciudad de Buenos Aires, Argentina.

^3^Department of Environmental Health Sciences, Istituto di Ricerche Farmacologiche Mario Negri IRCCS, Via Mario Negri 2, 20156, Milano, Italy

^#^Federico Fortuna and Alessia Lanno contributed equally to this work and shared first authorship.

^$^Alice Passoni and Renzo Bagnati share senior authorship

*Alice Passoni is the corresponding author.

alice.passoni@marionegri.it , Department of Environmental Health Sciences, Istituto di Ricerche Farmacologiche Mario Negri IRCCS, Via Mario Negri 2, 20156, Milano, Italy.

***Table S1. Reproducibility assessment of the analytical method using 13 replicates of a pooled sample from a full-scalp shave of a single donor.***

| Replicate | Calculated concentration pg/mg | Mean | SD | CV% |
| --- | --- | --- | --- | --- |
| 1 | 6.73 | 6.51 | 1.24 | 19% |
| 2 | 4.46 |  |  |  |
| 3 | 6.45 |  |  |  |
| 4 | 5.97 |  |  |  |
| 5 | 4.22 |  |  |  |
| 6 | 7.19 |  |  |  |
| 7 | 6.35 |  |  |  |
| 8 | 6.30 |  |  |  |
| 9 | 8.01 |  |  |  |
| 10 | 8.35 |  |  |  |
| 11 | 7.27 |  |  |  |
| 12 | 5.67 |  |  |  |
| 13 | 7.63 |  |  |  |

Results are expressed as mean, standard deviation (SD) and coefficient of variation (CV%).

***Table S2. Median Hair Cortisol (pg/mg) by Binary Variables (Mann–Whitney U test)***

| Variable | Group | Cortisol median (pg/mg) | IQR (p25–p75) | *p*-value |
| --- | --- | --- | --- | --- |
| Gender | Female | 2.33 | 1.57 – 3.27 | 0.296 |
|  | Male | 2.51 | 1.77– 3.41 |  |
| Tobacco use | Yes | 2.25 | 1.49 – 2.81 | 0.386 |
|  | No | 2.44 | 1.63 – 3.40 |  |
| Alcohol use | Yes | 2.42 | 1.61 – 3.35 | 0.689 |
|  | No | 2.39 | 1.57 – 3.41 |  |
| Physical activity | Yes | 2.52 | 1.70 – 3.45 | 0.117 |
|  | No | 2.18 | 1.51 – 3.15 |  |
| Hair dyeing | Yes | 2.34 | 1.47 – 3.05 | 0.294 |
|  | No | 2.51 | 1.78 – 3.44 |  |
| Chronic illness | Yes | 2.18 | 1.46 – 3.23 | 0.511 |
|  | No | 2.44 | 1.66 – 3.39 |  |
| Perceived stress | High (>23) | 2.53 | 1.95 – 3.43 | 0.117 |
|  | Low (<23) | 2.35 | 1.53 – 3.28 |  |

***Table S3. Median Hair Cortisol (pg/mg) by Multi-Category Variables (Kruskal–Wallis test)***

| Variable | Group (n) | Cortisol median (pg/mg) | IQR (p25–p75) | *p*-value |
| --- | --- | --- | --- | --- |
| Age | 18 – 34 (155) | 2.47 | 1.60 – 3.28 | 0.949 |
|  | 35- 44 (40) | 2.31 | 1.74 – 3.08 |  |
|  | 45- 59 (35) | 2.45 | 1.65 – 4.73 |  |
|  | > 60 (26) | 2.15 | 1.40 – 3.98 |  |
| BMI | Underweight (20) | 2.15 | 1.49 – 2.89 | 0.024 |
|  | Normal weight (180) | 2.28 | 1.53 – 3.09 |  |
|  | Overweight (46) | 2.93 | 1.80 – 4.13 |  |
|  | Obese (10) | 2.73 | 1.54 – 6.15 |  |
| Alcohol Consumption | 1 dose/week (97) | 2.28 | 1.52 – 3.17 | 0.465 |
|  | 2 doses/week (40) | 2.57 | 1.89 – 3.49 |  |
|  | 3 doses/week (21) | 2.27 | 1.52 – 3.09 |  |
|  | >3 doses/week (23) | 2.53 | 2.23 – 3.25 |  |
| Physical activity | 30 – 60 min/week (22) | 2.02 | 1.42 – 2.49 | 0.096 |
|  | 1 – 3 hours/week (64) | 2.59 | 1.59 – 3.29 |  |
|  | 3 – 5 hours/week (47) | 2.68 | 1.70 – 3.72 |  |
|  | ≥5 hours /week (22) | 2.52 | 2.12 – 3.76 |  |
| Hair washing | 1 time/week (13) | 2.62 | 2.00 – 3.45 | 0.925 |
|  | 2 times/week (79) | 2.47 | 1.91 – 3.41 |  |
|  | 3 times/week (92) | 2.41 | 1.76 – 3.57 |  |
|  | 4 times/week (35) | 2.24 | 1.67 – 3.01 |  |
|  | ≥5 times/week (37) | 2.51 | 1.52 – 3.80 |  |
| Occupation | Researcher (80) | 2.31 | 1.50 – 3.01 | 0.553 |
|  | PhD student (36) | 2.51 | 1.78 – 3.36 |  |
|  | Bachelor Student (53) | 2.47 | 1.96 – 3.33 |  |
|  | Administrative Staff (64) | 2.26 | 1.58 – 3.52 |  |
|  | Other (23) | 2.70 | 1.72 – 3.51 |  |

***Table S4.* Health Assessment and Demographic Questionnaire**

| **Category** | **Variable** | **Response Options / Scale** |
| --- | --- | --- |
| **Demographics** | Age | Continuous (years) |
|  | Gender | Male, Female, Other |
|  | Occupation | Open-ended |
| **Anthropometrics** | Height | Continuous (cm) |
|  | Weight | Continuous (kg) |
| **Health Status** | Do you consider yourself to be healthy | Yes, No |
|  | Do you have any chronic health condition? | None, Hypertension, Diabetes, Cardiovascular disease, Asthma, Others |
|  | Are you currently taking or using any medication?  Please specify any of the following:  Oral contraceptives (ACO) or hormonal replacement therapy.  Psychiatric or neurological treatments (e.g., antidepressants, anxiolytics, hypnotics, antipsychotics, or antiepileptics).  Topical treatments (creams or ointments), especially those containing corticosteroids.  Inhaled or nasal medications (e.g., inhalers for asthma or sprays for allergies).  Any other chronic or temporary medication | Yes (specify), No |
|  | Have you been hospitalized recently? (last six month) | Yes, No |
|  | Pregnancy | Yes,No |
| **Lifestyle & Habits** | Do you exercise regularly? | Yes (Frequency: 30-60 min/week, 1-3 h/week, 3-5 h/week, >5 h/week), No |
|  | Do you use tobacco? | Yes (Amount: <10, 10-20, 20-30, >30 cigarettes/day), No |
|  | Do you drink alcoholic beverages? | Yes (Frequency: 1, 2, 3, >3 drinks/week), No |
| **Hair Care** | What product do you use to wash your hair? | Shampoo, Shampoo and conditioner, None, Others |
|  | Do you have dyed hair?" | Yes, No |
|  | Weekly hair washing frequency | 1, 2, 3, 4, ≥5 times per week |

**S5. Perceived Stress Scale (PSS-10) Items**

The questions in this scale ask about your feelings and thoughts during the **last month.** In each case, you will be asked to indicate **how often you felt or thought** a certain way. The best approach is to answer fairly quickly. That is, don’t try to count up the number of times you felt a particular way; rather indicate the alternative that seems like a reasonable estimate.

(0 = Never, 1 = Almost never, 2 = Sometimes, 3 = Often, 4 = Very often)

Question: In the last month, how often have you...

1,...been upset because of something that happened unexpectedly?

2,...felt that you were unable to control the important things in your life?

3,...felt nervous and "stressed"?

4,...felt confident about your ability to handle your personal problems?

5,...felt that things were going your way?

6,...found that you could not cope with all the things that you had to do?

7,...been able to control irritations in your life?

8,...felt that you were on top of things?

9,...been angered because of things that were outside of your control?

10,...felt difficulties were piling up so high that you could not overcome them?
